# From single pathways to holistic networks: unraveling the multimechanistic actions of acupuncture in depression

**DOI:** 10.3389/fpsyt.2026.1936946

**Published:** 2026-10-23

**Authors:** Shaohao Quan, Wanyi Xu, Haofei Du, Zhifang Xu, Tianyi Zhao, Meidan Zhao, Xiaowei Lin, Xiaokai Chen

**Affiliations:** 1 Department of Rehabilitation Medicine, The Affiliated Huizhou Hospital of Guangzhou Medical University, Huizhou, China; 2 Tianjin Key Laboratory of Modern Chinese Medicine Theory of Innovation and Application, School of Traditional Chinese Medicine, Tianjin University of Traditional Chinese Medicine, Tianjin, China

**Keywords:** acupuncture, depression, electroacupuncture, neuroinflammation, pain comorbidity

## Abstract

Depression is a globally prevalent mental disorder; however, current pharmacological treatments are limited by a delayed onset of action and significant adverse effects. As a safe and effective non-pharmacological therapy, acupuncture demonstrates unique advantages in treating mild to moderate depression. Its antidepressant mechanisms are multifaceted. Primarily, acupuncture mitigates neuroinflammation by inhibiting classical signaling pathways (e.g., nuclear factor kappa B/NOD-like receptor family pyrin domain containing 3), modulating microglial polarization, and restoring cytokine homeostasis. Furthermore, it enhances neuroplasticity by upregulating brain-derived neurotrophic factor expression, improving synaptic plasticity, and promoting adult neurogenesis. At the cellular level, acupuncture exerts neuroprotective effects by suppressing apoptotic cascades associated with mitochondrial dysfunction and endoplasmic reticulum stress, regulating autophagic balance, and restoring neuronal excitability. Systemically, it ameliorates metabolic dysregulation by optimizing cerebral energy metabolism, modulating gut microbiota composition, and reinforcing intestinal barrier integrity. Additionally, acupuncture effectively alleviates comorbid pain by modulating peripheral nociceptive signaling and central pain-emotion feedback loops. These synergistic effects across multiple systems and targets may together ameliorate the pathophysiological alterations associated with depression. Despite these advances, current research faces challenges, including the translational gap between animal models and clinical pathology, as well as an insufficient investigation of multi-mechanism interactions. Future efforts should focus on developing clinically relevant models, integrating multi-omics technologies, conducting high-quality clinical trials, and exploring biomarker-guided personalized treatments to advance the scientific and standardized application of acupuncture for depression.

## 1 Introduction

Depression is a widespread and debilitating mental health disorder globally, and it is increasingly affecting younger populations, resulting in profound losses in productivity and substantial economic burdens (1, 2). Recent epidemiological data indicate that over 264 million individuals worldwide suffer from depression, and suicide triggered by major depressive disorder has become the leading cause of suicide globally (3). However, the current clinical landscape for depression treatment remains suboptimal. Although chemically synthesized antidepressants, such as selective serotonin reuptake inhibitors, are the first-line treatment, they are associated with significant inter-individual variability, limited overall efficacy, and various adverse effects, including dizziness (4). Consequently, there is an urgent need to identify complementary therapies that offer fewer side effects and higher patient acceptability.

Acupuncture, a therapeutic modality deeply rooted in traditional medicine, has demonstrated immense potential and unique advantages in alleviating depressive symptoms (5). Numerous clinical trials and systematic reviews consistently suggest that acupuncture, whether administered as a monotherapy or an adjunct to conventional treatments, effectively mitigates depressive symptoms with a minimal risk of adverse events (6–9). Nevertheless, the primary obstacle to establishing acupuncture as a universally accepted, evidence-based treatment in modern medicine is the incomplete elucidation of its underlying mechanisms. Early mechanistic studies primarily focused on the regulation of monoamine neurotransmitters (10). With rapid advancements in neuroscience, immunology, and microbiomics, research has expanded to encompass gene transcription, cellular microenvironments, and systemic metabolic networks (11, 12). Extensive preclinical and clinical studies have confirmed that acupuncture at specific acupoints can robustly modulate the central nervous system (CNS), regulate immune-endocrine responses, and coordinate systemic metabolism (13–15).

Given the vast accumulation of basic research data, the field urgently needs to address several core scientific questions: Does acupuncture exert its antidepressant effects through a “common molecular hub” rather than through disparate, isolated pathways? What are the hierarchical and interactive relationships among these diverse mechanisms? To this end, this review systematically synthesizes recent basic research findings on acupuncture for depression. We construct a comprehensive mechanistic framework encompassing five key dimensions: suppression of neuroinflammation, promotion of neuroplasticity, regulation of neuronal apoptosis and activity, alleviation of comorbid pain, and modulation of metabolism. This review aims to provide a robust biological rationale for the clinical application of acupuncture and to outline future translational directions for mechanistic research.

## 2 Methods

We comprehensively searched three major biomedical databases—PubMed, Web of Science, and Embase—from their inception to April 2026. The search terms included (“acupuncture” OR “electroacupuncture” OR “EA” OR “MA”) AND (“depression” OR “psychogenic”). A total of 4,376 records were initially identified. After the first round of screening, 3,518 records—including reviews, meta-analyses, and studies irrelevant to the research question or lacking a preclinical/clinical study design—were excluded, leaving 858 records. After excluding 735 duplicate records, a total of 123 eligible studies were included in this review. The literature screening process is summarized in **Figure 1**.

**Figure 1:**
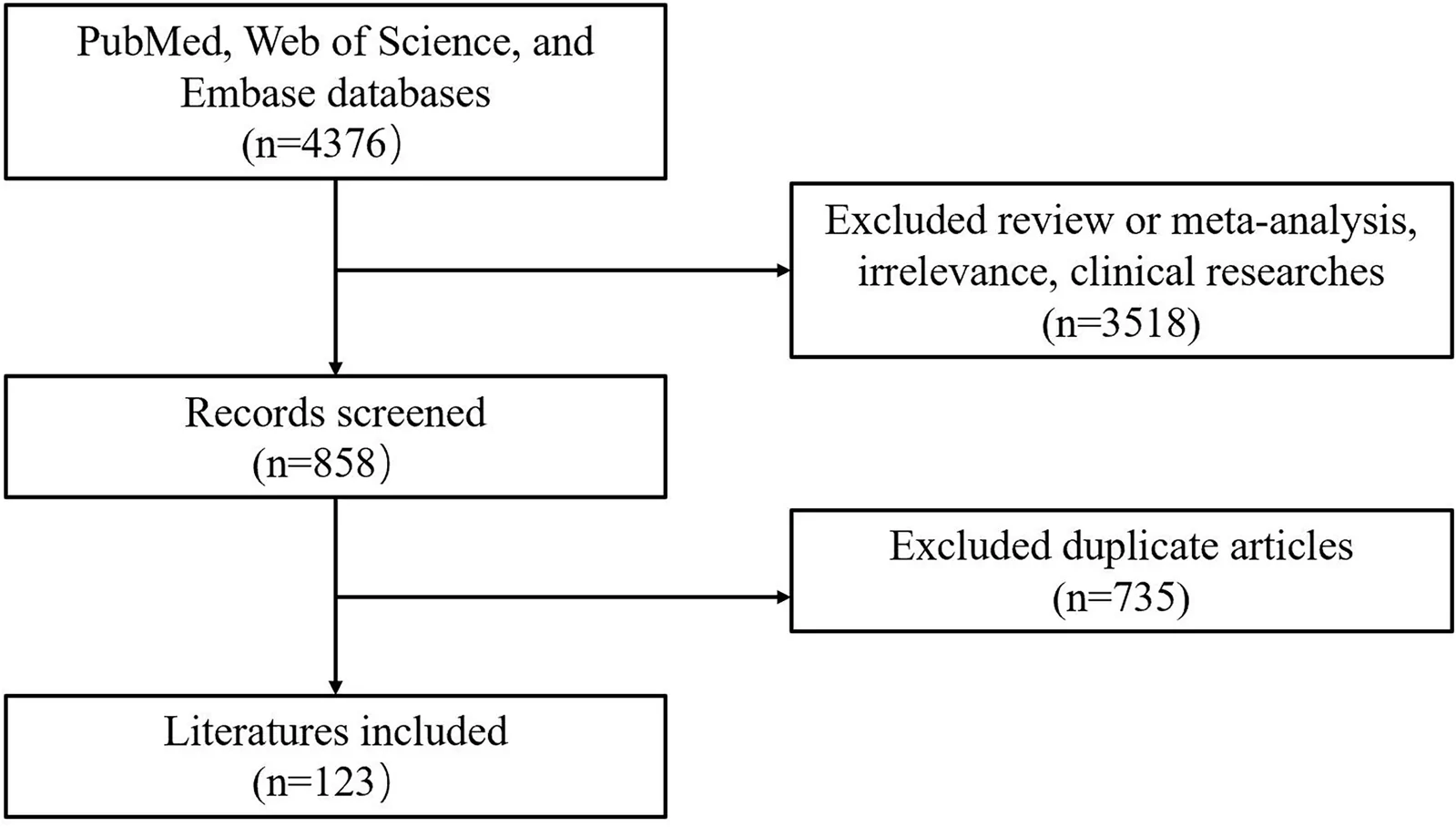
Flowchart of literature retrieval.

## 3 The mechanism of acupuncture in treating depression

This section systematically elucidates the specific biological mechanisms underlying acupuncture treatment for depression across five key domains: suppression of inflammatory responses, promotion of neuroplasticity, regulation of neuronal apoptosis and activity, alleviation of comorbid pain, and regulation of metabolism (**Figure 2**).

**Figure 2:**
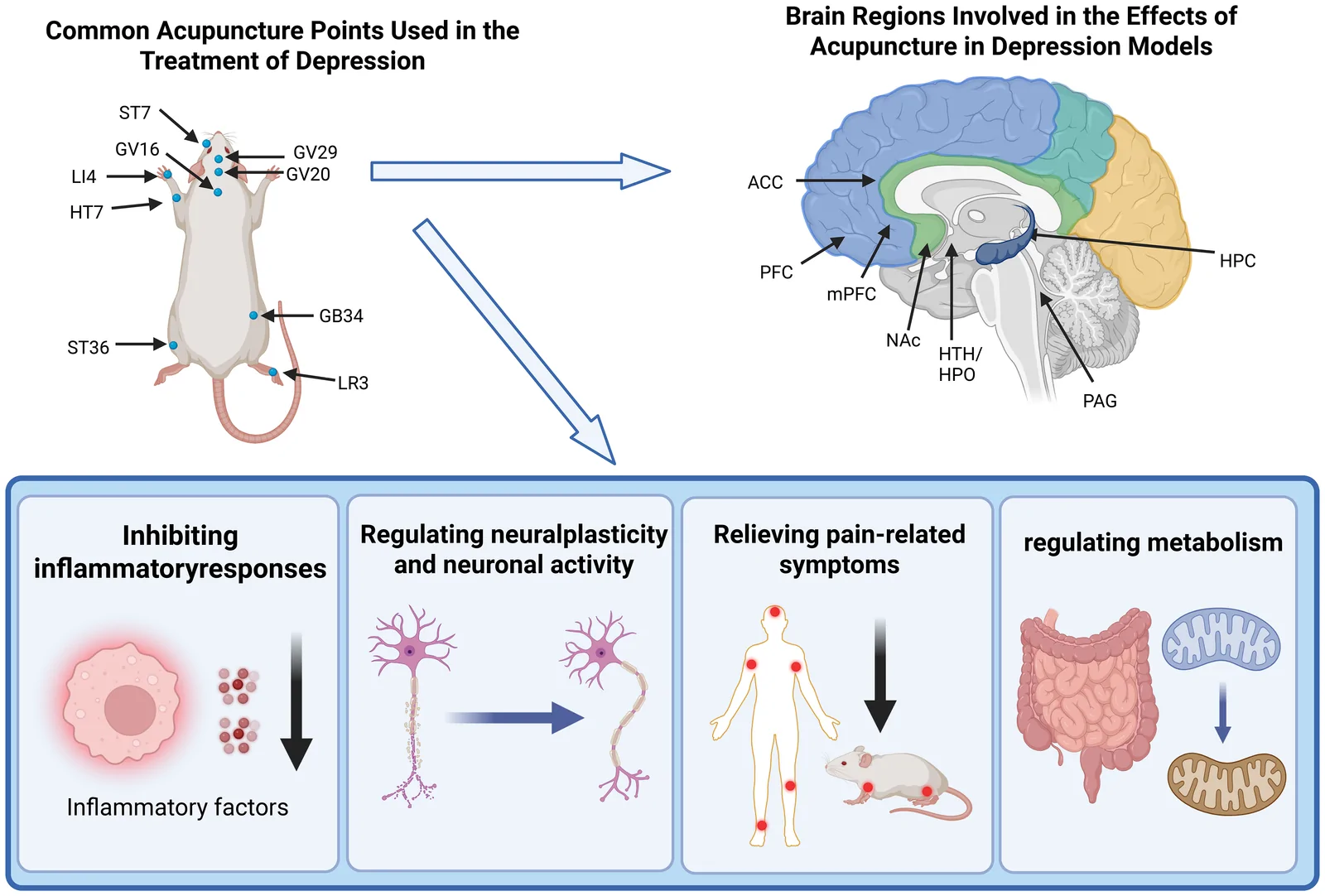
Biological mechanisms of acupuncture in the treatment of depression. These mechanisms include suppressing inflammatory responses, promoting neuroplasticity and regulating neuronal activity, alleviating pain, and regulating metabolism. ACC, anterior cingulate cortex; PFC, prefrontal cortex; mPFC, medial prefrontal cortex; NAc, nucleus accumbens; HTH/HPO, hypothalamus/hypothalamic–pituitary–ovarian axis; PAG, periaqueductal gray; HPC, hippocampus. Created in BioRender. Xu, W. (2026) https://BioRender.com/ieuc5rg.

### 3.1 Acupuncture alleviates depression by suppressing neuroinflammation

In recent years, the complex bidirectional relationship between neuroinflammation and depression has emerged as a focal point in neuroscience. Neuroinflammation is essentially an innate immune defense response of the CNS, typically characterized by the abnormal activation of microglia and astrocytes, accompanied by elevated local levels of pro-inflammatory cytokines and chemokines (16). During the pathogenesis of depression, prolonged pathological stress initially activates the peripheral immune system, triggering the release of inflammatory cytokines. These peripheral inflammatory signals can penetrate the CNS through a compromised blood–brain barrier (BBB), thereby inducing and amplifying neuroinflammation (17). This persistent inflammatory microenvironment exhibits potent neurotoxicity, directly disrupting neurotransmitter metabolism and severely impairing neural plasticity, ultimately driving the onset and consolidation of depressive symptoms (18). Acupuncture has been extensively validated as an effective anti-inflammatory intervention. Its core mechanism involves precisely inhibiting key inflammatory signaling pathways, reprogramming glial cell phenotypes, and restoring cytokine network homeostasis, thereby halting the inflammatory cascade at its source and fostering a favorable microenvironment for neuroplasticity recovery.

#### 3.1.1 Balancing the network of pro- and anti-inflammatory cytokines

A growing body of evidence suggests that an imbalance in peripheral and central cytokine networks is a critical driver of depression pathology (19). Following prolonged stress exposure, pro-inflammatory cytokines such as IL-1β, IL-6, and TNF-α are abnormally elevated (11). These signals not only interfere with monoamine neurotransmitter synthesis but also compromise BBB integrity (11, 20, 21). Increased BBB permeability allows peripheral immune cells and inflammatory mediators to infiltrate the CNS directly, further exacerbating neuroinflammation (22). Therefore, restoring cytokine network homeostasis is a pivotal therapeutic strategy.

Numerous studies have demonstrated that acupuncture can serve as a systemic immunomodulatory intervention that influences cytokine networks. First, acupuncture can effectively suppress the release of pro-inflammatory cytokines. In a lipopolysaccharide (LPS)-induced acute immune stress model, Zhang et al. (23) found that electroacupuncture (EA) at GV20 and GV29 significantly reduced the serum and hippocampal levels of IL-1β, IL-6, and TNF-α while simultaneously downregulating indoleamine 2,3-dioxygenase (IDO) activity and the concentration of neurotoxic quinoline acid. In a chronic restraint stress (CRS) model, Guo et al. (24) demonstrated that EA pretreatment significantly downregulated pro-inflammatory factors in the hippocampal CA3 region, exhibiting preventive antidepressant effects comparable to the classic antidepressant imipramine.

Beyond merely suppressing pro-inflammatory factors, acupuncture emphasizes a “bidirectional balance” within the inflammatory network and neuroendocrine feedback regulation. Zheng et al. (25) reported that in a perimenopausal depression (PMD) model, acupuncture at GV20, BL23, BL18, and SP6 not only reduced inflammatory factors but also simultaneously increased the levels of the anti-inflammatory cytokine IL-10. This bidirectional regulation is closely associated with the restoration of the hypothalamic–pituitary–ovarian (HPO) axis function and the activation of the phosphatidylinositol 3-kinase (PI3K)/protein kinase B (AKT)/mammalian target of rapamycin (mTOR) signaling pathway.

#### 3.1.2 Modulating classic inflammatory signaling pathways

Nuclear factor kappa B (NF-κB), a “key transcription factor” regulating inflammatory responses, plays a crucial role in depression pathology through its cascading activation with the NOD-like receptor family pyrin domain-containing 3 (NLRP3) inflammasome; this axis is a core target of acupuncture. A 2024 study by Tong et al. (14) using a chronic unpredictable mild stress (CUMS) rat model revealed that EA at GV23 and GV16 significantly upregulated GABA_B receptor expression in the lateral habenula (LHb), thereby inhibiting the NF-κB/NLRP3 inflammatory pathway. This led to a reduced expression of NLRP3, IL-1β, and IL-6 while elevating IL-10, ultimately ameliorating depressive-like behaviors. Reverse validation using the GABA_B-specific antagonist CGP55845 completely abolished these effects, confirming the GABA_B-NF-κB/NLRP3 axis as a critical pathway for EA’s anti-inflammatory action.

In addition to directly targeting the NLRP3 inflammasome, acupuncture exerts broad negative regulation on NF-κB through upstream signaling cascades (e.g., the PI3K/AKT pathway). Guo et al. (24) demonstrated that EA pretreatment at classic emotion-regulating acupoints (GV20 and GV29) in a CRS model significantly suppressed the abnormal hyperactivation of the NF-κB pathway in the hippocampal CA3 region. This finding not only further solidifies acupuncture’s role as a regulatory target for NF-κB-related pathways but also provides a deeper explanation for how acupuncture can preemptively eliminate potential inflammation, thereby preventing depressive-like behaviors before they arise.

#### 3.1.3 Regulating the polarization balance of microglia

At the cellular level, acupuncture’s regulation of CNS-resident immune cells—particularly microglia and astrocytes—has garnered significant attention. An imbalance in microglial polarization (i.e., excessive activation of the pro-inflammatory M1 phenotype and suppression of the anti-inflammatory M2 phenotype) is central to neuroinflammation (26). Under chronic stress, microglia tend to polarize toward the M1 phenotype, releasing massive amounts of pro-inflammatory factors (27, 28). Extensive research confirms that acupuncture restores microglial polarization balance through multiple mechanisms (29–32).

In depression models with diverse pathophysiological backgrounds, acupuncture demonstrates multidimensional strategies for regulating glial cells. In the CRS model, Chen et al. (31) found that early acupuncture at the GV20 and GV29 inhibits the upstream expression and release of high mobility group box 1 (HMGB1) in the hippocampus, thereby preventing excessive microglial activation. Yue et al. (30) further demonstrated in a chronic unpredictable stress (CUS) model that EA downregulates the purinergic receptor P2X7, inhibiting the activation of NLRP3 inflammasome, which not only reverses pathological microglial activation but also alleviates astrocyte atrophy. Conversely, in a post-stroke depression (PSD) model, acupuncture focuses on promoting the “phenotypic conversion” of microglia. Studies by Li et al. (33) and Rao et al. (29) showed that EA suppresses hyperactive Toll-like receptor 4 (TLR4)/p38/NF-κB/NLRP3 and PI3K/AKT/NF-κB signaling pathways in PSD and middle cerebral artery occlusion (MCAO) models. This not only reduces Iba-1 and pro-inflammatory cytokine expression but, more importantly, promotes the conversion of M1 microglia to the tissue-repairing M2 phenotype, creating a favorable microenvironment for hippocampal neurogenesis. Furthermore, acupuncture at HT7 curbs M1 phenotypic conversion by upregulating endogenous protective substances such as neuropeptide Y (NPY) (**Figure 3**) (34).

**Figure 3:**
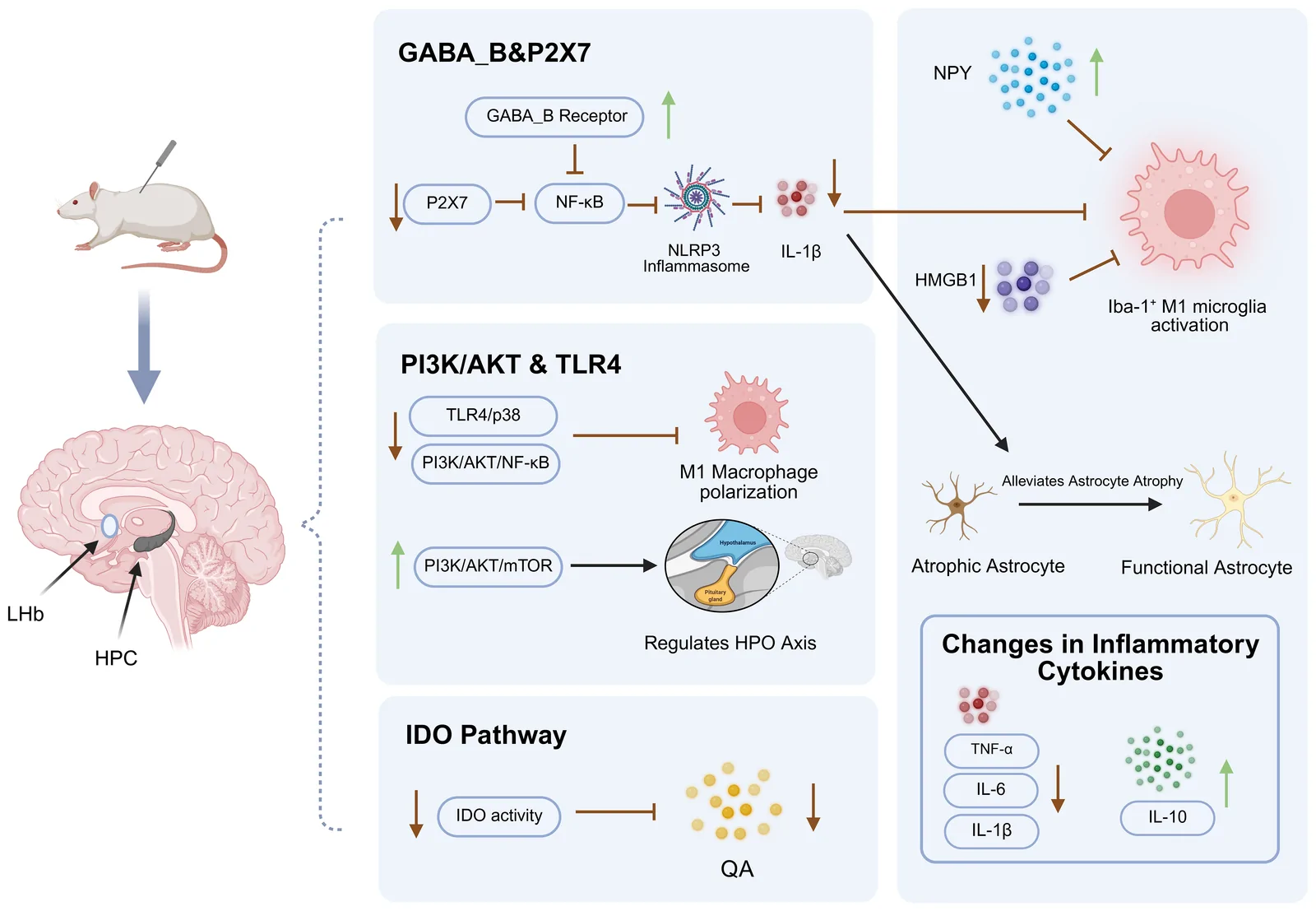
Acupuncture suppresses neuroinflammation by modulating inflammatory cytokines, signaling pathways, and microglial polarization. Acupuncture can alleviate neuroinflammation in the LHb and HPC brain regions. Specifically, acupuncture upregulates the expression of GABA_B receptors in these brain regions and downregulates the expression of purinergic ligand-gated ion channel 7 (P2X7) on microglia, thereby inhibiting the NF-κB/NLRP3 inflammatory pathway and reducing the release of pro-inflammatory cytokines. Acupuncture also significantly inhibits the TLR4/p38 and PI3K/AKT/NF-κB signaling pathways to reduce Iba-1 expression. Simultaneously, by suppressing HMGB1 release and upregulating NPY, it collectively inhibits excessive microglial activation and alleviates astrocyte atrophy. Acupuncture downregulated IDO activity and the concentration of the neurotoxic quinoline acid and activated the PI3K/AKT/mTOR signaling pathway to improve depressive symptoms. LHb, lateral habenula; IDO, indoleamine 2,3-dioxygenase; QA, quinoline acid; HMGB1, high mobility group box 1; NPY, neuropeptide Y. Created in BioRender. Xu, W. (2026) https://biorender.com/quu38xi.

### 3.2 Acupuncture combats depression by enhancing neuroplasticity

Impaired neuroplasticity is considered the core microstructural basis of depression (35). The nervous system’s ability to adapt to changes in the internal and external environment through structural and functional remodeling is severely compromised under prolonged pathological stress (36). A wealth of evidence indicates that acupuncture acts as a “potent modulator” of neuroplasticity (37–39). This section elucidates the biological mechanisms by which acupuncture reverses depression-related neuroplasticity abnormalities, focusing on three key aspects: promoting the expression of neurotrophic factors, regulating synaptic structure and function, and enhancing neurogenesis.

#### 3.2.1 Promoting the expression and signaling of neurotrophic factors

Brain-derived neurotrophic factor (BDNF) and its high-affinity receptor tropomyosin receptor kinase B (TrkB) constitute a classic central pathway regulating neuroplasticity (40). In models such as CUMS, EA interventions (e.g., at GV20 and GV29) significantly upregulate BDNF gene and protein expression in key brain regions such as the hippocampus and prefrontal cortex (PFC), enhancing TrkB phosphorylation (41, 42). This activation triggers two key downstream signaling cascades: the PI3K/AKT and MEK/ERK signaling pathways, where the PI3K/AKT pathway primarily mediates neuronal survival and homeostasis, while the MEK/ERK pathway drives synaptic function and neurogenesis (43, 44).

Acupuncture exerts acupoint- and brain-region-specific effects on BDNF. Needling GV20 primarily upregulates BDNF expression in the hippocampus (42, 45), whereas needling HT7 has a more pronounced effect on BDNF regulation in the PFC (46). ST36, conversely, exhibits broad regulatory effect on BDNF across multiple brain regions (47). This specificity may relate to differences in the neuroanatomical pathways and central projection areas associated with each acupoint.

Beyond BDNF, acupuncture regulates the expression of other neurotrophic factors. Lim et al. (34) found that acupuncture at HT7 and ST36 significantly upregulates NPY in the hippocampus in a maternal–infant separation model. As a vital neuroprotective factor, NPY indirectly promotes neuroplasticity by suppressing excessive stress responses and reducing neuronal damage. Furthermore, acupuncture upregulates basic fibroblast growth factor (FGF2) and nerve growth factor (NGF), further elucidating the mechanisms by which acupuncture modulates the neurotrophic factor network (48, 49).

#### 3.2.2 Parameter-dependent mechanistic characteristics of acupuncture: acupoint specificity and electroacupuncture frequency effects

Pre-clinical studies have adopted heterogeneous acupoint prescriptions and EA parameters, yet how these intervention settings shape downstream biological outputs remains incompletely systematized (50). Existing animal evidence suggests partial mechanistic preference for certain acupoint combinations and distinct frequency-dependent neurochemical responses.

Acupoint combinations exhibit partial target preference for inflammation, neuroplasticity, or pain-related pathways. Acupoint pair GV20 + GV29 produces prominent anti-neuroinflammatory and pro-neurogenesis effects, mainly targeting hippocampal neuroinflammation and neuroplastic remodeling (31). Acupuncture at HT7 shows biased modulation of prefrontal-limbic emotional circuits and is more closely associated with mood-relevant neuroplastic regulation (34). ST36 acts as a multi-target acupoint, capable of regulating both peripheral inflammatory responses and gut–brain–axis metabolic homeostasis (51). For pain-depression comorbidity models, the GV20 + ST36 combination is frequently deployed, which concurrently modulates peripheral nociceptive signaling and limbic emotional networks (52). Nevertheless, well-powered head-to-head comparative studies for most acupoint formulae are still scarce; it remains unclear whether such preferences are reproducible across different depression animal models (50). This represents a critical knowledge gap for future mechanism-oriented acupuncture research.

Low- and high-frequency EA differentially engage opioid, GABAergic, and monoaminergic systems. Low-frequency EA (2 Hz) preferentially activates enkephalin- and β-endorphin-related opioid signaling and exerts stronger modulatory influences on serotonergic and dopaminergic monoaminergic pathways (53). In contrast, high-frequency EA (100 Hz) predominantly mobilizes dynorphin-dependent opioid pathways and yields more robust regulation of spinal and supraspinal GABAergic inhibitory neurotransmission (54). Mixed-frequency EA can theoretically activate multiple opioid receptor subtypes simultaneously, but its unique mechanistic advantages require further experimental validation.

Notably, most of the above-mentioned parameter-mechanism correlations derive from rodent pre-clinical work; high-quality clinical evidence directly linking acupoint/frequency settings to human biological readouts is still insufficient (55). Standardized comparative trials are warranted to guide parameter optimization for clinical antidepressant acupuncture practice.

#### 3.2.3 Improving synaptic structural and functional plasticity

Synaptic plasticity is a core manifestation of neural plasticity. Morphological studies show that depression models exhibit significant reductions in synaptic density, abnormal widening of the synaptic cleft, and thinning of the PSD in the hippocampal CA1 and dentate gyrus (DG) (56, 57). Jia et al. observed via transmission electron microscopy that EA at GV20 and ST7 2 weeks later effectively reversed the aforementioned structural damage and promoted the restoration of normal morphology at the protrusion interface (57).

Functionally, acupuncture optimizes synaptic transmission efficiency. A 2024 study by Li et al. (58) found that EA at GV23 and GV16 upregulated postsynaptic density protein 95 (PSD95) and synaptic vesicle protein (SYP) in the lateral region of the raphe nucleus while downregulating the expression of N-methyl-D-aspartate receptors (NMDARs). Jiang et al. (59) found that EA at LI4 and LR3 modulates the subunit composition and phosphorylation status of AMPA receptors (AMPARs), thereby enhancing the long-term potentiation (LTP) effect of synaptic transmission. These structural and functional restorations are highly dependent on the upstream cAMP–PKA–CREB signaling pathway. The use of the PKA inhibitor H89 significantly blocks these EA-induced effects, confirming the pathway’s indispensability (60, 61).

#### 3.2.4 Promoting neurogenesis

Adult neurogenesis is primarily concentrated in the subgranular zone (SGZ) and the subventricular zone (SVZ) (62). Its abnormal arrest directly disrupts the stability of the hypothalamic–pituitary–adrenal (HPA) axis and impairs emotional and cognitive functions, directly leading to the core symptoms of depression (63, 64). In the CUMS model, (65, 66) EA intervention increased the number of BrdU-labeled newborn neurons in the hippocampal SGZ and improved the survival and differentiation rate.

Mechanistically, acupuncture promotes neurogenesis by (1) upregulating BDNF/TrkB signaling to directly drive neural progenitor cell proliferation, differentiation, and migration, (41, 67) (2) suppressing CNS inflammation, thereby eliminating the toxic inhibitory effects of inflammatory factors and providing a healthy microenvironment for cell regeneration, (68) and (3) maintaining neural progenitor pool stability via Notch and Wnt/β-catenin signaling pathways (69). Remarkably, acupuncture exhibits cell-type-specific regulation: it promotes the division of amplifying neural progenitors (ANPs) while protecting quiescent neural progenitors (QNPs) from apoptosis (70).

### 3.3 Acupuncture combats depression by regulating neuronal homeostasis

The onset of depression is closely associated with increased neuronal apoptosis and abnormal neuronal activity in emotion-regulating brain regions (71–74). This section discusses how acupuncture exerts neuroprotective effects through inhibiting apoptotic pathways, regulating autophagic balance, and restoring neuronal activity.

#### 3.3.1 Inhibiting neuronal apoptosis

Apoptosis is a genetically regulated form of programmed cell death that normally plays a role in clearing excess cells during nervous system development; when neurons in the adult brain are subjected to sustained damage, this pathway is abnormally activated, leading to neuronal loss (75). Studies have shown that in various models of depression (such as CUMS and PSD), neurons exhibit characteristic morphological changes, including nuclear condensation (76). Acupuncture precisely targets and inhibits multiple key pathways that trigger apoptosis, thereby exerting a potent neuroprotective effect (77).

The mitochondrial apoptosis pathway is one of the primary targets of acupuncture regulation. EA sat LI4, GV29, and GV20 significantly upregulates the anti-apoptotic protein B-cell lymphoma 2 (Bcl-2) in the hippocampus and downregulates the pro-apoptotic protein Bcl-2-associated X protein (Bax), thereby reducing the Bax/Bcl-2 ratio. This effectively inhibits the release of cytochrome C and the subsequent activation of caspase-9 and caspase-3 (25, 77–80). Regarding endoplasmic reticulum (ER) stress, pathological stress causes misfolded protein accumulation, triggering the unfolded protein response (81, 82). Shen et al. (83) demonstrated that EA effectively downregulates the expression of key molecules in the protein kinase R-like endoplasmic reticulum kinase (PERK)–eukaryotic translation initiation factor 2α (eIF2α)–activating transcription factor 4 (ATF4)–C/EBP homologous protein (CHOP) pathway and cleaved caspase-12, blocking the ER stress-mediated apoptotic pathway (84, 85). Furthermore, with regard to the death receptor pathway triggered by exogenous inflammatory factors (such as TNF-α) (86), acupuncture indirectly inhibits caspase-8 activation by reducing pro-inflammatory cytokines, thereby attenuating its amplifying effect on mitochondrial apoptotic signaling (33, 87).

Acupuncture’s anti-apoptotic intervention is not limited to isolated protein inhibition but constitutes a synergistic network. By upregulating the BDNF and the PI3K/Akt survival pathway, it achieves a “triple containment” of the mitochondrial, ER, and death receptor apoptosis pathways, providing a solid molecular foundation for neuroprotection.

#### 3.3.2 Improving neuronal homeostasis by regulating autophagic balance

Autophagy is vital for maintaining intracellular homeostasis (88). In the depressive microenvironment, neurons often exhibit excessive autophagic activation, leading to pathological “self-consumption” (89, 90). Acupuncture exerts its antidepressant effects by regulating autophagy balance and improving the neuronal survival environment (91, 92).

Zhang et al. (91) found that the hippocampal CA1 region in CUMS rats exhibited excessive autophagy (increased autolysosomes, elevated microtubule-associated protein 1 light chain 3 (LC3)-II/LC3-I ratio, and upregulated BCL2 interacting coiled-coil protein 1 (Beclin1)). EA at GV20 and GV29 significantly suppressed this excessive autophagy, restoring markers to physiological levels. The adenosine 5′-monophosphate-activated protein kinase (AMPK)/mTOR energy-sensing pathway serves as the central hub. Zheng et al. (25) indicated that acupuncture significantly downregulates AMPK phosphorylation levels while upregulating mTOR and p70S6K activity, exerting a negative regulatory signal on the autophagy-initiating molecule Unc-51-like autophagy-activating kinase 1 (ULK1).

#### 3.3.3 Restoring neuronal activity and circuit function

Abnormal neuronal activity is an important functional change in depression, manifested as abnormal discharge frequency and functional connectivity changes of neurons in emotion-regulating brain regions (93). Acupuncture restores normal neural circuit function through multiple pathways (94).

Regulating membrane potential and ion balance is fundamental. Hippocampal neurons in depression models exhibit reduced resting membrane potentials and decreased excitability (95). EA restores normal membrane potentials by upregulating voltage-gated sodium (Nav1.1) and potassium (Kv4.2) channels, increasing the amplitude and frequency of excitatory postsynaptic potentials (EPSPs) (96). Concurrently, EA maintains intracellular calcium homeostasis, preventing calcium-overload-induced neurotoxicity (97, 98).

At the receptor level, EA upregulates (γ-aminobutyric acid B) GABA_B receptors in the LHb to enhance GABAergic inhibition and reduce pathological hyperexcitability (58, 99). In the hippocampus, EA modulates NMDAR and AMPAR function to improve glutamatergic synaptic transmission (100). Functional magnetic resonance imaging (fMRI) studies provide *in vivo* evidence: patients with depression exhibit overactivation of the default mode network (DMN) and reduced connectivity in the salience network (SN). EA treatment significantly normalizes these network aberrations, correcting abnormal neuronal activity in emotion-regulating brain regions (101, 102) (**Figure 4**).

**Figure 4:**
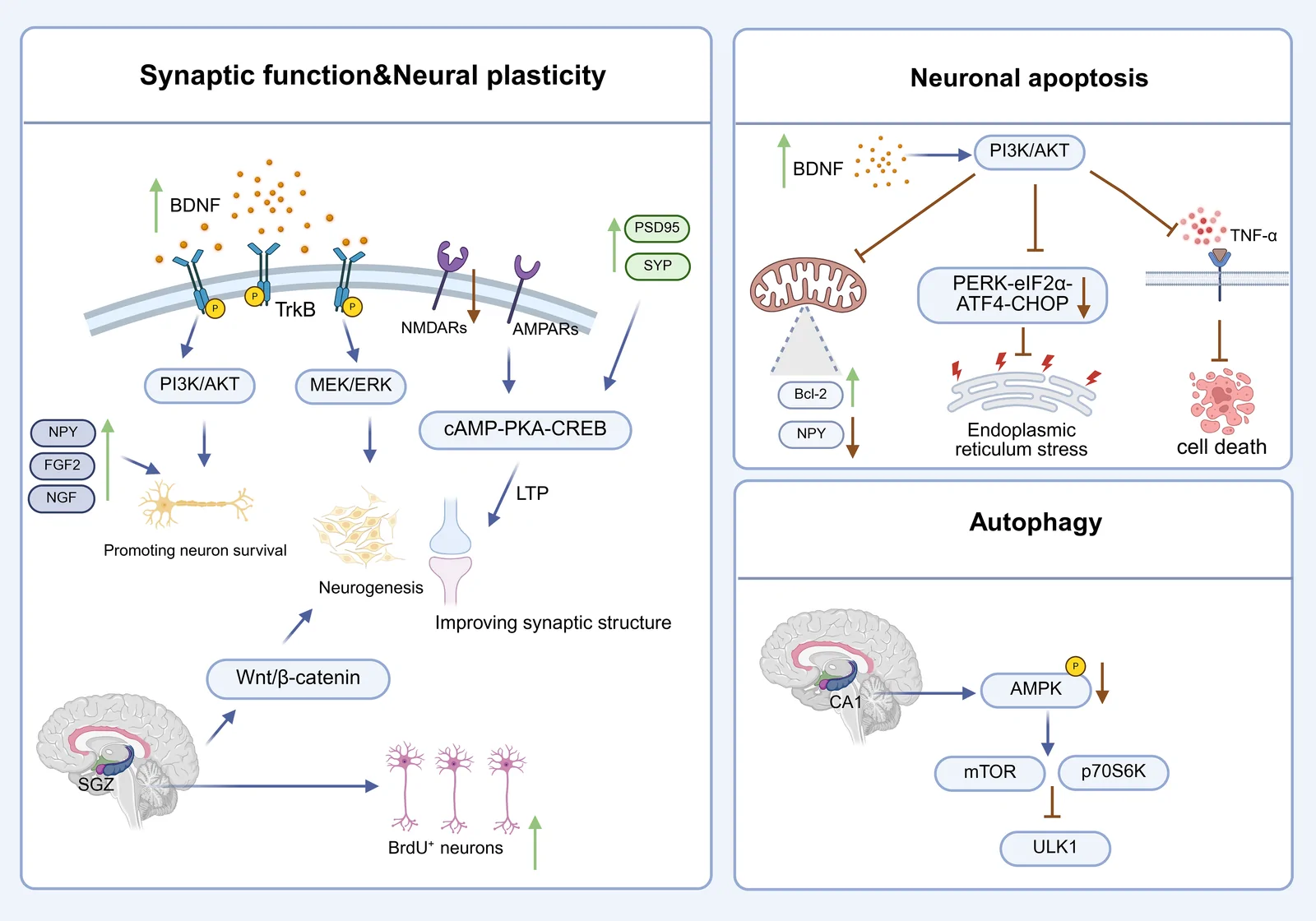
Acupuncture improves depression by enhancing neural plasticity and regulating neuronal homeostasis. Acupuncture upregulates BDNF expression to modulate the PI3K/AKT and MEK/ERK signaling pathways. The PI3K/AKT pathway primarily mediates neuronal survival and homeostasis, exerting a triple inhibitory effect on mitochondria, the endoplasmic reticulum, and the death receptor-mediated apoptotic pathway, while the MEK/ERK pathway drives synaptic function and neurogenesis. In addition, acupuncture upregulates the expression of NPY, NGF, and FGF2 to collectively promote neuronal plasticity. By upregulating the expression of PSD95 and SYP, downregulating NMDARs, and modulating the subunit composition and phosphorylation status of AMPARs, acupuncture enhances the LTP of synaptic transmission; these structural and functional recoveries are highly dependent on the upstream cAMP–PKA–CREB signaling pathway. Acupuncture maintains the stability of the neural progenitor cell pool via the Wnt/β-catenin signaling pathway while also increasing the number of BrdU-labeled newborn neurons in the SGZ. Acupuncture also significantly downregulates AMPK phosphorylation levels while upregulating the activity of mTOR and p70S6K, thereby exerting a negative regulatory signal on the autophagy-initiating molecule ULK1 and improving neuronal homeostasis. BDNF, brain-derived neurotrophic factor; NMDARs, N-methyl-D-aspartate receptors; AMPARs, α-amino-3-hydroxy-5-methyl-4-isoxazolepropionic acid receptors; PSD95, postsynaptic density protein 95; SYP, synaptic vesicle protein; FGF2, fibroblast growth factor; NGF, nerve growth factor; SGZ, subgranular zone; Bcl-2, B-cell lymphoma 2; CA1, cornu ammonis 1; ULK1, Unc-51 like autophagy activating kinase 1; LTP, long-term potentiation. Created in BioRender. Xu, W. (2026) https://biorender.com/xor7ay3.

### 3.4 Acupuncture alleviates depression by improving metabolic disorders

Metabolic abnormalities play a pivotal role in the occurrence and development of depression, including energy metabolism abnormalities, gut microbiota metabolic imbalance, and other aspects (103). Acupuncture regulates metabolic disorders through multiple targets and pathways to improve depressive symptoms (104).

#### 3.4.1 Maintains neural function by improving energy metabolism

Abnormal energy metabolism, characterized by reduced cerebral glucose metabolism and mitochondrial dysfunction, is a key pathological feature of depression (105). Acupuncture exerts its antidepressant effects by improving energy metabolism, ensuring an adequate energy supply to neurons, and maintaining normal cellular function (106).

Restoring cerebral glucose metabolism is a primary mechanism underlying these effects. Positron emission tomography (PET) reveals that depressed patients exhibit reduced glucose metabolism in the PFC and hippocampus, correlating with symptom severity (107, 108). In rat models, EA interventions reverse these metabolic deficits by upregulating glucose transporters (HK and GLUT3), thereby accelerating glucose uptake into neurons and glial cells (109, 110). Furthermore, EA modulates the activity of key glycolytic enzymes, such as hexokinase and phosphofructokinase, promoting the aerobic oxidation of glucose and enhancing adenosine triphosphate (ATP) production efficiency (111).

Protecting mitochondrial function is the second key mechanism (112). As the primary energy-generating organelles, mitochondrial deficits severely impair central energy metabolism in depression. In CUMS models, EA significantly improves mitochondrial dysfunction in the hippocampus, evidenced by stabilized mitochondrial membrane potential, increased ATP production, reduced reactive oxygen species (ROS) generation, and inhibited mitochondrial permeability transition pore (mPTP) opening (56, 113, 114). Mechanistic studies indicate that EA promotes mitochondrial biogenesis by upregulating key transcriptional regulators, including PGC-1α, NRF1, and TFAM (56). Concurrently, it bolsters the mitochondrial antioxidant defense system by enhancing the expression and activity of superoxide dismutase (SOD) and glutathione peroxidase (GSH-Px), thereby mitigating oxidative-stress-induced organelle damage (115).

The regulation of the AMPK/mTOR energy-sensing axis underpins these metabolic improvements (116). AMPK serves as a critical sensor of cellular energy status, and its excessive activation is a hallmark of energy deficiency (117). In depression models, elevated AMPK phosphorylation and concomitantly reduced mTOR activity in the hippocampus lead to suppressed protein synthesis and metabolic dysregulation (92). EA effectively dampens AMPK phosphorylation while restoring mTOR activity, thereby rebalancing energy-sensing networks and supporting neuronal function (118). Additionally, in PMD models, acupuncture indirectly modulates the AMPK/mTOR axis via upstream activation of the PI3K/AKT pathway, further ameliorating metabolic deficits (25).

#### 3.4.2 Regulating the gut–brain axis via microbiota metabolism

Dysregulation of the gut–brain axis is increasingly recognized as a key pathophysiological mechanism underlying depression. Multiple studies using the CUMS rat model have consistently shown that the emergence of depression-like behavior is closely associated with significant dysbiosis of the gut microbiota (66, 119–121). This dysbiosis is primarily characterized by a loss of overall microbial diversity and pathological shifts in the relative abundance of specific phyla and genera.

Modulating the composition of the gut microbiota is the foundation for acupuncture’s ability to improve gut microbiota metabolism. EA interventions at acupoints such as GV20, GV29, ST36, and ST25 correct microbial imbalances by enriching beneficial probiotics (e.g., *Bifidobacterium* and *Streptococcus*) and suppressing pathogenic taxa (119, 120). 16S rRNA sequencing confirms that EA mainly improves the abnormal microbiota structure by regulating the α-diversity (e.g., Chao1 and Shannon indices) and β-diversity of the gut microbiota (51, 121). The regulation of gut microbiota metabolites is an important pathway through which acupuncture influences the function of the gut–brain axis (122). Short-chain fatty acids (SCFAs), tryptophan metabolites, and bile acids produced by gut microbiota metabolism can influence the function of the CNS through the blood circulation and neural pathways (123)—for instance, serum and fecal SCFA levels (acetate, propionate, and butyrate) are significantly depleted in depressed rats; EA treatment robustly replenishes these metabolites, with a particularly marked elevation in butyrate (124). As a primary energy substrate for colonocytes, butyrate not only reinforces the intestinal barrier but also crosses the BBB to modulate neuroinflammation and neuroplasticity, exerting profound antidepressant effects (123).

Preserving intestinal barrier integrity is fundamental to acupuncture’s regulation of the gut–brain axis. Animal models of depression exhibit significant impairment of intestinal barrier function, characterized by downregulated tight junction proteins (ZO-1, occludin, and claudin-4), elevated intestinal permeability, and subsequent endotoxemia (125, 126). EA significantly upregulates these tight junction proteins, seals the intestinal barrier, and prevents the systemic translocation of endotoxins like LPS. This blockade effectively blunts systemic inflammation and neuroinflammation (127). Furthermore, EA reduces circulating biomarkers of intestinal permeability, including diamine oxidase (DAO), D-lactic acid (D-LA), and LPS-binding protein (LBP), confirming its potent barrier-protective properties (119) (**Figure 5**).

**Figure 5:**
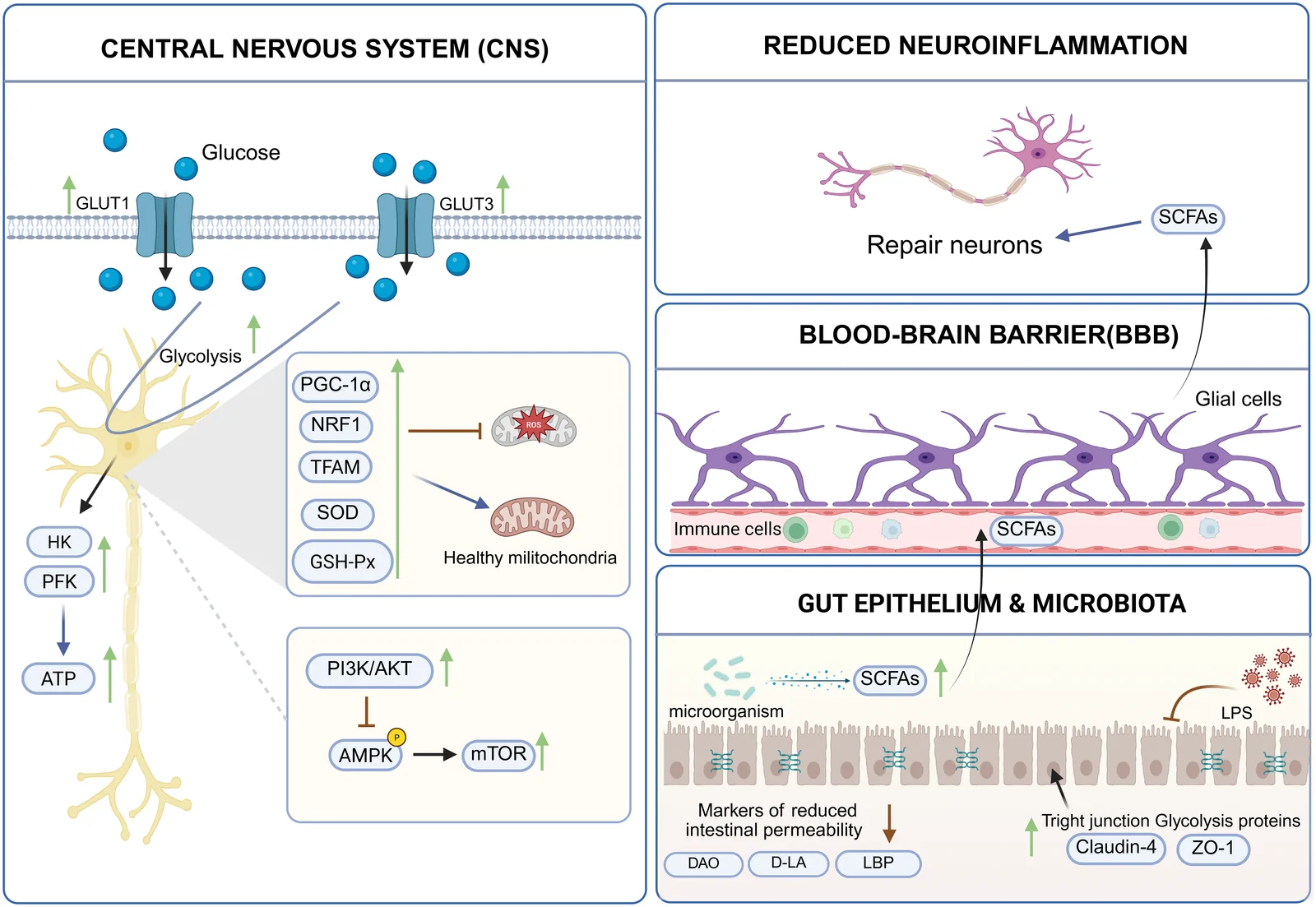
Metabolic mechanisms by which acupuncture improves depression. Acupuncture exerts its antidepressant effects by improving energy metabolism, ensuring that neurons receive an adequate energy supply and maintain normal cellular function. Acupuncture upregulates glucose transporters to accelerate glucose uptake by neurons and glial cells. In addition, acupuncture modulates the activity of key glycolytic enzymes, such as hexokinase and phosphofructokinase, to promote the aerobic oxidation of glucose and enhance ATP production. Acupuncture promotes mitochondrial biogenesis by upregulating key transcriptional regulators such as PGC-1α, NRF1, and TFAM. It also enhances the expression and activity of superoxide dismutase and glutathione peroxidase, thereby strengthening the mitochondrial antioxidant defense system and reducing organelle damage. Acupuncture also effectively inhibits AMPK phosphorylation and restores mTOR activity, thereby rebalancing energy sensing networks and supporting neuronal function. Furthermore, acupuncture significantly upregulates intestinal tight junction proteins, reduces circulating biomarker levels associated with intestinal permeability, repairs the intestinal barrier, and prevents the systemic translocation of endotoxins such as lipopolysaccharides, effectively suppressing systemic and neuroinflammation. Modulating the composition of the gut microbiota is the foundation for acupuncture’s ability to improve gut microbial metabolism. Acupuncture effectively supplements SCFAs. SCFAs not only strengthen the intestinal barrier but also cross the blood–brain barrier to modulate neuroinflammation and neuroplasticity, thereby exerting profound antidepressant effects. GLUT1/GLUT3, glucose transporters; HK, hexokinase; PFK, phosphofructokinase; SOD, superoxide dismutase; GSH-Px, glutathione peroxidase; DAO, diamine oxidase; D-LA, d-lactic acid; LBP, LPS-binding protein; SCFAs, short-chain fatty acids. Created in BioRender. Xu, W. (2026) https://biorender.com/8av6jv3.

## 4 Mechanisms of acupuncture in alleviating pain–depression comorbidity

Pain and depression frequently co-occur, driving a bidirectional vicious cycle. On the one hand, chronic pain acts as a persistent stressor that triggers depressive symptoms by activating neuroinflammatory responses, leading to the depletion of monoamine neurotransmitters, and impairing the function of the prefrontal–limbic system circuit in the brain (128). Conversely, depression exacerbates pain perception by downregulating central endogenous analgesics (e.g., endorphins), heightening pain sensitivity, and distorting cognitive coping strategies (129). This complex bidirectional mechanism often limits the effectiveness of single-dimensional treatments. As an effective method for pain management, acupuncture offers unique advantages in addressing pain-related depression (130). This section elucidates how acupuncture concurrently ameliorates pain and depression by modulating nociceptive signaling, rectifying pain–emotion circuit interactions, and regulating neurotransmitter networks.

### 4.1 Acupuncture interferes with pain signal transmission and integration

The aberrant generation, amplification, and processing of nociceptive signals form the pathophysiological substrate linking pain and depression. By synergistically modulating the peripheral–spinal–central pain pathways, acupuncture reduces pain input, alleviates pain symptoms, and subsequently improves depressive mood.

At the peripheral level, acupuncture directly suppresses nociceptor hyperexcitability. In a pain–depression comorbidity model induced by chronic infraorbital nerve (CION) compression (CION), EA at GV20 and ST36 significantly reduced peripheral neuropathic discharges activity and diminished the transmission of pain signals to the CNS (32). These peripheral effects are mediated by two primary mechanisms: upregulating endogenous opioid peptides (e.g., endorphins and enkephalins) to activate peripheral opioid receptors (131) and inhibiting nociceptive ion channels, such as transient receptor potential vanilloid 1 (TRPV1), to attenuate inflammatory hyperalgesia (132).

At the spinal cord level, acupuncture blunts nociceptive amplification by reinforcing endogenous inhibitory neurotransmitter systems (133, 134). Specifically, EA upregulates the expression of GABA_A and GABA_B receptors in the spinal dorsal horn, thereby enhancing GABA- and glycine-mediated inhibitory effects (135). Simultaneously, it recruits descending noradrenergic and serotonergic (5-HT) pathways, exerting dual inhibitory control over dorsal horn projection neurons (136, 137).

At the supraspinal level, acupuncture modulates neuronal activity in key brainstem analgesic centers, including the dorsal raphe nucleus (DRN) and periaqueductal gray (PAG), to bolster descending pain modulation (138–140). EA rectifies functional aberrations in higher-order pain-processing cortical regions—such as the PFC, insula, and anterior cingulate cortex (ACC)—thereby mitigating the negative affective dimension of pain (141). Clinical functional imaging confirms that EA attenuates ACC hyperactivation in patients with comorbid pain and depression, effectively dampening the emotional amplification of nociception (52).

### 4.2 Modulating pain–emotion circuit interactions

The therapeutic efficacy of acupuncture relies heavily on dismantling the vicious cycle linking pain and emotion. Chronic pain transcends mere sensory perception; it serves as a potent stressor that chronically activates affective neural circuits, thus precipitating depressive symptoms. Conversely, a depressive state amplifies nociception by lowering pain thresholds, heightening sensitivity, and inducing hypervigilance toward pain (142). Acupuncture disrupts this feedback loop by precisely targeting key brain regions and neural circuits governing pain–emotion integration.

The ACC serves as a critical hub for integrating nociception with affective responses, and its dysfunction is central to pain-related depression (143). Under comorbid conditions, ACC neurons exhibit hyperexcitability and pathologically enhanced synaptic transmission, directly driving negative emotional experiences and the affective amplification of pain (144). EA treatment markedly suppresses this neuronal hyperexcitability and normalizes aberrant functional connectivity between the ACC and interconnected regions, such as the amygdala and PFC (145, 146). Molecular mechanism studies have shown that EA enhances serotonergic inhibition by upregulating 5-HT1A receptors in the ACC while concurrently modulating the BDNF/TrkB pathway to restore structural and functional neuroplasticity (78, 141).

The amygdala, as the center of emotional processing, becomes hyperactive under pain-induced stress. Specifically, excitation of the central nucleus of the amygdala (CeA) drives HPA axis hyperactivity and systemic inflammation, exacerbating anxiety and depression (147). Acupuncture effectively suppresses CeA neuronal overactivation, curbing the release of pro-inflammatory cytokines and stress hormones. Simultaneously, it enhances the inhibitory regulatory tone of the basolateral amygdala (BLA), thereby correcting aberrant emotional processing (148, 149).

The dopamine (DA) system is instrumental in acupuncture improving pain-related anhedonia. Patients with pain-related depression are accompanied by obvious anhedonia frequently, which is closely related to the abnormal function of the DA system (150). EA significantly elevates DA levels within reward-circuit regions, including the ventral tegmental area (VTA) and nucleus accumbens, thereby restoring reward processing and alleviating anhedonic symptoms (150). Concurrently, restored DA signaling enhances acupuncture’s analgesic efficacy via the descending modulation of nociceptive sensitivity.

In summary, by precisely modulating affective hubs like the ACC, amygdala, and reward circuitry, acupuncture successfully uncouples the emotional dimension of pain from its sensory transmission. This network-level regulation constitutes the neural circuit basis for its dual efficacy in alleviating both pain and depression (151) (**Figure 6**).

**Figure 6:**
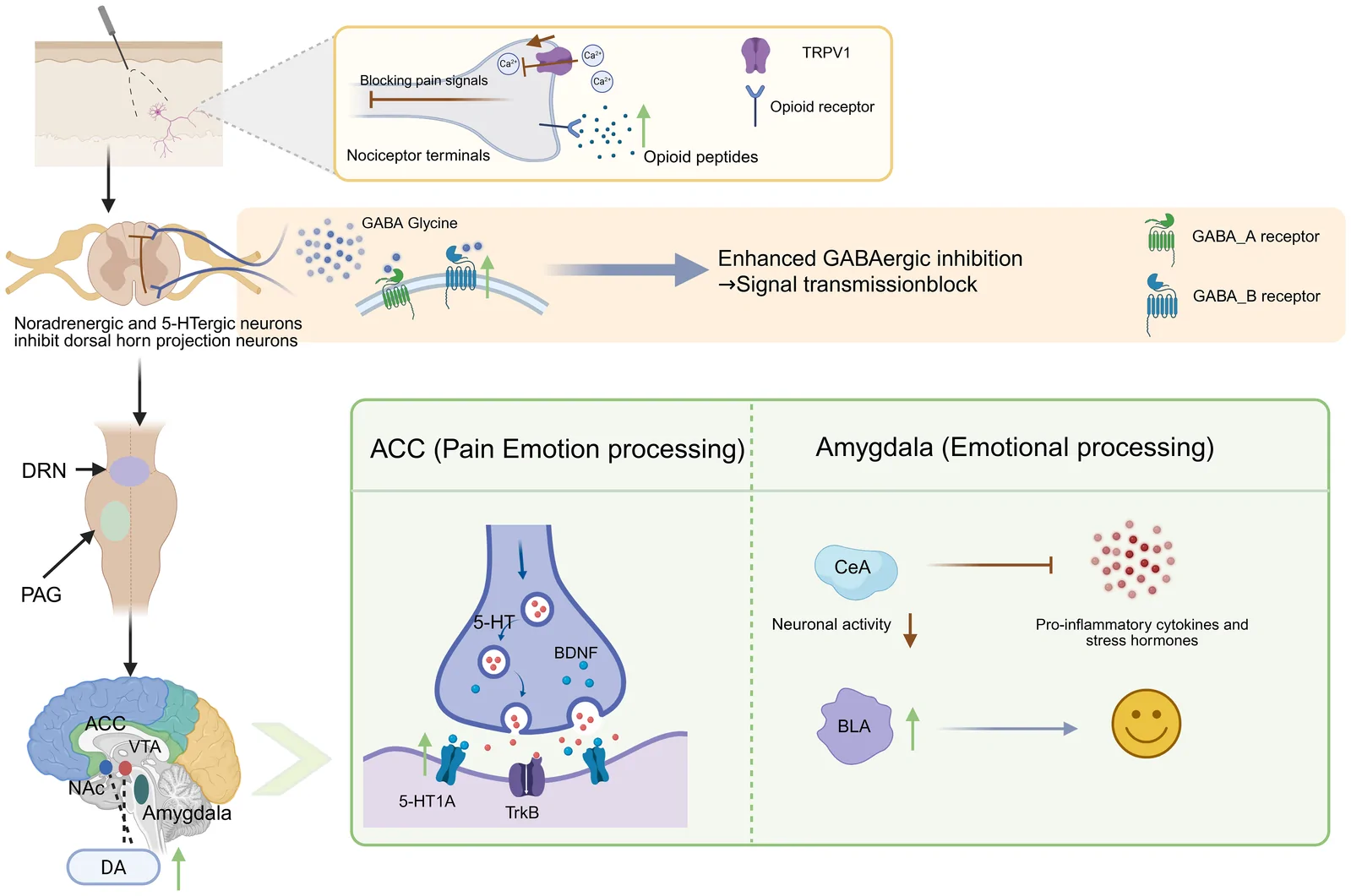
Mechanism by which acupuncture improves depression involves pain relief. Acupuncture can upregulate peripheral endogenous opioid peptide levels and activate peripheral opioid receptors, as well as inhibit pain-sensing ion channels such as TRPV1 at nociceptor terminals, thereby alleviating inflammatory hyperalgesia. Acupuncture enhances GABA- and glycine-mediated inhibitory effects by upregulating the expression of GABA_A and GABA_B receptors in the dorsal horn of the spinal cord. Concurrently, acupuncture modulates descending noradrenergic and serotonergic pathways, exerting dual inhibitory regulation on projection neurons in the dorsal horn of the spinal cord to suppress pain amplification. Acupuncture also modulates neuronal activity in the brainstem’s key analgesic centers, the DRN and PAG, thereby enhancing descending pain modulation. Furthermore, acupuncture enhances serotonergic inhibition by upregulating 5-HT1A receptors in the ACC while simultaneously modulating the BDNF/TrkB pathway to restore the plasticity of neural structure and function. Acupuncture also effectively inhibits the excessive activation of CeA neurons, thereby suppressing the release of pro-inflammatory cytokines and stress hormones. At the same time, it enhances the inhibitory modulatory role of the BLA, thereby correcting abnormal emotional processing. Finally, acupuncture significantly increases dopamine levels in the VTA and NAc, thereby restoring reward processing functions and alleviating the symptoms of anhedonia. TRPV1, transient receptor potential V1; DRN, dorsal raphe nucleus; PAG, periaqueductal gray; VTA, ventral tegmental area; DA, dopamine; CeA, central nucleus of the amygdala; BLA, basolateral amygdala. Created in BioRender. Xu, W. (2026) https://biorender.com/z8ey28w.

## 5 Discussion

In summary, the core advantage of acupuncture in treating depression lies not in the potent modulation of a single target but, rather, in its capacity to orchestrate multilevel and systemic synergistic regulation. The five core mechanisms delineated in this review—suppression of neuroinflammation, enhancement of neuroplasticity, regulation of neuronal apoptosis and activity, alleviation of pain comorbidity, and amelioration of metabolic disorders—do not operate in isolation. They instead form a highly intertwined and complementary regulatory network that spans the nervous, immune, endocrine, and metabolic systems. These coordinated actions together reverse the pathophysiological aberrations of depression and restore systemic homeostasis (55, 152). To further illustrate this network-level crosstalk, key bridging molecular nodes across these dimensions are summarized below.

Multiple shared molecular hubs serve as critical bridges interconnecting the five mechanistic dimensions. BDNF/TrkB acts as a central mediator: besides driving neuroplastic remodeling and adult neurogenesis, BDNF/TrkB signaling also inhibits neuroinflammatory cascades, improves neuronal anti-apoptotic capacity, modulates pain–emotion circuit plasticity, and participates in gut–brain axis metabolic feedback (40, 42, 60). The PI3K/AKT/mTOR axis exerts broad pleiotropic effects: it restrains NF-κB-driven neuroinflammation, supports neuronal survival and synaptic plasticity, balances autophagic flux, and mediates gut-microbiota-derived metabolite signaling (25, 29, 43, 44). The NF-κB/NLRP3 inflammasome is not merely an inflammatory executor; its activity can be modulated by neurotrophic signaling, autophagy status, and metabolic perturbations, further propagating pathological signals to impair neuroplasticity and amplify pain-related negative affect (14, 24). AMPK, the cellular energy sensor, couples cerebral energy metabolism with autophagy, microglial polarization, and neurotrophic factor transcription, integrating metabolic status with immune-neuronal responses (91, 116, 117). SCFAs represent peripheral–central messengers: they reinforce intestinal barrier integrity, suppress systemic inflammation, cross the blood–brain barrier to modulate microglial phenotypes, and promote hippocampal neurogenesis (123, 124). Collectively, these hub molecules enable crosstalk among seemingly separate biological processes and underpin the network–based antidepressant property of acupuncture.

Despite remarkable progress in elucidating the antidepressant mechanisms of acupuncture, several limitations in current research warrant attention. First, a translational gap exists between animal models and clinical practice; existing models fail to fully recapitulate the etiological complexity of human depression, including psychosocial stressors, comorbidities, and inter-individual variability. Second, although mechanistic investigations have spanned multiple biological levels, the majority remain confined to isolated signaling pathways or specific brain regions. There is a lack of comprehensive exploration regarding the crosstalk among these mechanisms and their network-level spatiotemporal dynamics. Third, clinical translation remains inefficient, as the transformation of basic research findings into standardized, evidence-based clinical protocols lacks systematic validation. Finally, the framework for personalized acupuncture therapy is still in its infancy, with precision intervention strategies tailored to specific depression subtypes, sex differences, and varying treatment responses remaining largely exploratory.

Future research should focus on breakthroughs in the following directions. First, more clinically relevant disease models should be developed, such as gender-specific models, multifactorial comorbid models, and refractory depression models, to improve the clinical relevance of research. Second, multi-omics technologies (genomics, transcriptomics, metabolomics, etc.) should be integrated with multimodal imaging techniques to elucidate the molecular and brain network mechanisms of acupuncture regulation from a systems biology perspective. Third, high-quality clinical research and advance multicenter, large-sample, and long-term follow-up randomized controlled trials should be conducted, with enhanced quantitative correlation analysis between therapeutic mechanisms and clinical efficacy (153). Fourth, biomarker-guided personalized treatment protocols and precision matching of acupoint selection and therapeutic parameters should be achieved by combining core acupoints (e.g., GV20 and GV29) identified through network biology research. Fifth, international collaboration and standardization efforts should be strengthened to promote the international integration of mechanistic research, therapeutic efficacy evaluation systems, and clinical application norms for acupuncture in depression treatment (55).

Driven by technological innovations and multidisciplinary integration, the core antidepressant mechanisms of acupuncture will be more systematically elucidated, thereby enhancing the scientific rigor and precision of its clinical application. Ultimately, this will not only provide more sufficient evidence-based support for the widespread clinical promotion of acupuncture but also offer new ideas and strategies for the diversified treatment of depression, ultimately benefiting more patients with the disorder.
